# Dental water jet efficacy in the plaque control of orthodontic patients wearing fixed appliance: A randomized controlled trial 

**DOI:** 10.4317/jced.55411

**Published:** 2019-11-01

**Authors:** Sergio Mazzoleni, Alberto De Stefani, Carola Bordin, Paolo Balasso, Giovanni Bruno, Antonio Gracco

**Affiliations:** 1 Associate Professor, Department of Neuroscience, University of Padua. Via Giustiniani 2 - 35100 – Padua (Italy); 2 DDS, Department of Neuroscience, University of Padua. Via Giustiniani 2 - 35100 – Padua (Italy); 3 Dental Hygienist, Department of Neuroscience, University of Padua. Via Giustiniani 2 - 35100 – Padua (Italy); 4 Department of Engineering and Management, University of Padua. Via Giustiniani 2 - 35100 – Padua (Italy)

## Abstract

**Background:**

Different studies assess the role of fixed orthodontic appliances in supragingival plaque accumulation. In patients wearing fixed orthodontic appliances a good management of oral hygiene is required in order to prevent complication like as decay, enamel demineralization, gingivitis, gingival hyperplasia and periodontitis. The aim of this Randomized Controlled Trial (RCT) is to evaluate the efficacy of the use of a DWJ in patients under orthodontic treatment with fixed multibracket appliance.

**Material and Methods:**

The study design was single-blinded RCT with a split mouth protocol. Each patient followed a personal cleaning protocol using a DWJ in addition to traditional brushing only on one side while just brushing on the control side. The side on which was decided to use the DWJ was chosen randomly and the dental hygienist who took the measurements was blind. Plaque and gingival indexes were evaluated at baseline and at one, three and six-months follow-up.

**Results:**

It did not emerge any difference in the plaque and gingival indexes trend between the two groups. Patients initially reported an worsening of the indexes at one month evaluation, then they set at baseline levels at three and six months.

**Conclusions:**

The dental water jet does not improve significantly the efficacy of home oral hygiene in orthodontic patients wearing a multi-bracket fixed appliance. Patients did not show the traditional worsening during the whole orthodontic therapy.

** Key words:**Dental water jet, fixed orthodontics, plaque index, gingival index.

## Introduction

It is widely demonstrated that bacterial plaque is the main etiological factor for periodontal disease ranging from marginal gingival inflammation to severe periodontitis. Different studies assess the role of fixed orthodontic appliances in supragingival plaque accumulation. Even if the periodontal condition may revert to baseline once the appliance is removed, it is fundamental not to determine irreversible damage to the periodontal support (1).

In patients wearing fixed orthodontic appliances a good management of the oral hygiene is required in order to prevent complication such as decay, enamel demineralization, gingivitis, gingival hyperplasia and periodontitis. In fact, several studies demonstrated that fixed orthodontic appliance makes the oral hygiene more difficult determine a consequent plaque accumulation around bands, brackets and archwires. A fixed appliance may also change the bacterial composition, reduce self-clean process, facilitate the bacterial plaque retention and can provoke gingival inflammation or enamel decalcification with soft tissue recession and teeth abrasion (1-3).

Different devices are used to maintain an adequate oral hygiene in orthodontic patients starting with manual or electric toothbrush associated with toothpaste, up to dental floss and brushes for the interproximal hygiene. Some hygienists also propose the use of the dental water jet (DWJ) for the oral hygiene.

A review regarding the DWJ benefits on oral hygiene evidenced a reduction of gingival inflammation, bleeding and pathogenic bacteria in various patients, such as patients in a supportive periodontal maintenance program, patients with implants, crowns or bridges, patients with diabetes (4-12). Low evidence on the efficacy of DWJ in the oral hygiene control on orthodontic patients is actually present in literature determining the need of studying the effect of this device on an orthodontic patients sample.

The aim of this Randomized Controlled Trial (RCT) is to evaluate the efficacy of the use of a domiciliary DWJ in patients under orthodontic treatment with fixed multibracket appliance.

## Material and Methods

The study design was single-blinded RCT with a split mouth protocol. In detail each patient followed a domiciliary cleaning protocol using a DWJ in addition to traditional brushing only on one side while just brushing on the control side. The side on which was decided to use the DWJ was chosen randomly and the dental hygienist who took the measurements was blind.

For this research a sample of twenty people was recruited, ten females and ten males, selected from the patients requiring an orthodontic evaluation at our dental department. The age ranged from 13 and 32 years old.

The patients, after a first examination and full complete records collection, started the orthodontic treatment with a multibracket fixed appliance (Victory Series Low Profile 3M Unitek) and MBT prescription. The patient sample included subjects that required an orthodontic fixed appliance, in general good health and a complete dentition.

Patients with systemic diseases, clefts, hypodontia, previous teeth extraction, periodontal problems or mental deficiency, smoking patients and non-compliant patients were excluded from the study. Moreover, patients that required teeth extraction during the orthodontic treatment were excluded from the study.

Patient’s baseline periodontal status was evaluated before the bonding. A skilled dental hygienist evaluated the Silness and Loe plaque index (PI) and Silness and Loe gingival index (GI).

-Parameters evaluated 

Plaque index

A value is assigned to each tooth surface: vestibular, mesial, lingual and distal. The assigned score to every surface is between 0 and 3 and it defines the plaque evaluated in the cervical third of the tooth.

0: absence of plaque.

1: the plaque is not visible to the naked eye and it is evaluable only after the passage of the periodontal probe on tooth surface.

2: plaque accumulation visible to the naked eye.

3: abundant plaque accumulation

Gingival index

A value is assigned to each tooth surface: vestibular, mesial, lingual and distal. The periodontal probe is used with a light pressure on the gingival sulcus to evaluate the bleeding.

The assigned score to every surface is between 0 and 3 and it defines the level of gingival inflammation.

0: healthy gingiva

1: light inflammation, with a soft color changing and little edema.

2: moderate inflammation, with color changing, tissue edema and bleeding on the probe.

3: severe inflammation. Intensive color changing and tissue edema and spontaneous bleeding.

Study design

The dental hygienist evaluated plaque index and gingival index and motivated the patients to a correct home oral hygiene using a DWJ in addition to traditional brushing. An orthodontist followed the patients for all the orthodontic treatment and randomly assigned the side on which the patients have to use the DWJ. The dental hygienist was blind and she did not know the side in which the DWJ was used for all the treatment.

The study was carried out in four stages:

- T0: before the bonding the dental hygienist evaluated PI and GI, then the bonding procedure was made by the orthodontist. At the end of the bonding the dental hygienist explained and motivated the patients to a correct daily domiciliary oral hygiene. All the patients used the GUM Sunstar Ortho toothbrush. Modified Bass technique was explained to the patients to improve the oral hygiene. A fluorine toothpaste was delivered to the patients and explained to use in association with the toothbrush. The use of dental floss Superfloss Oral-B and brush teeth GUM Trav-ler with a 0.9 mm diameter was explained to the patients. Moreover, the dental hygienist explained to the patients the use of DWJ Philips Sonicare Airfloss once a day in the evening with a video support. A written oral hygiene protocol was delivered to the patient to summarise how to use all the oral hygiene instrumentation. At the end of this procedure, the orthodontist randomly assigned the side in which the patients had to use the DWJ.

- T1: a month after the bonding procedure the dental hygienist evaluated the PI and GI and motivated the patients to the correct home oral hygiene. All the oral hygiene procedures were explained again to the patients.

- T2: three months after the bonding procedure the dental hygienist evaluated the PI and GI and motivated the patients to the correct home oral hygiene. All the oral hygiene procedures were explained again to the patients.

- T3: six months after the bonding procedure the dental hygienist evaluated the PI and GI and motivated the patients to the correct home oral hygiene. All the oral hygiene procedures were explained again to the patients.

Statistical analysis

Descriptive analysis using means and standard deviations was performed to find out a significant difference for what concerns the Gengival Index (GI), and Plaque Index (PI) in two different groups (standard cleaning, waterjet treatment) consisting of 29 patient per group. These measures have been monitored in three different sides of the patients’ month: considering incisive and canine teeth (GI.IC and PI.IC), premolars (GI.P and PI.P) and molars (GI.M and GI.M). The goal of the study was to figure out whether the measures previously described of two groups were significantly different or not in each time period (T0, T1, T2, T3). Statistical analysis was performed with the computing environment R version 3.2.1 to verify whether the measures of the two groups were significantly different for each time period. We also evaluated whether each measure mean was significantly different across the three time periods. In particular, for the first case we used the t-test with Welch correction while for the second one the paired t-test with Welch correction was performed. Since the variances of the sample we want to compare are not the same ( Table 1) we use this correction that is particularly indicated in a context of heterogeneity of variances.

**Table 1:**
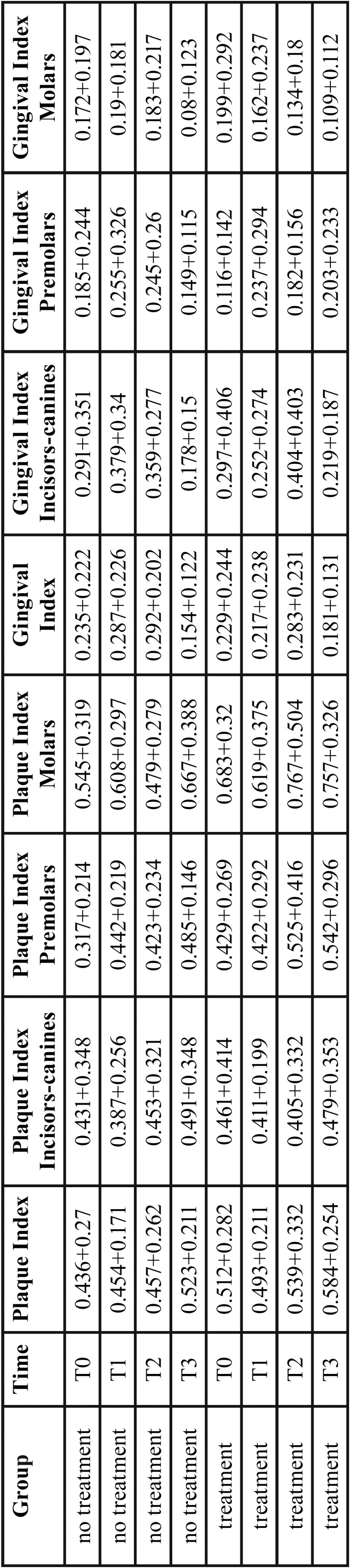
Means and standard deviations of plaque and gingival indexes per group and time period.

## Results

**Figure 1:**
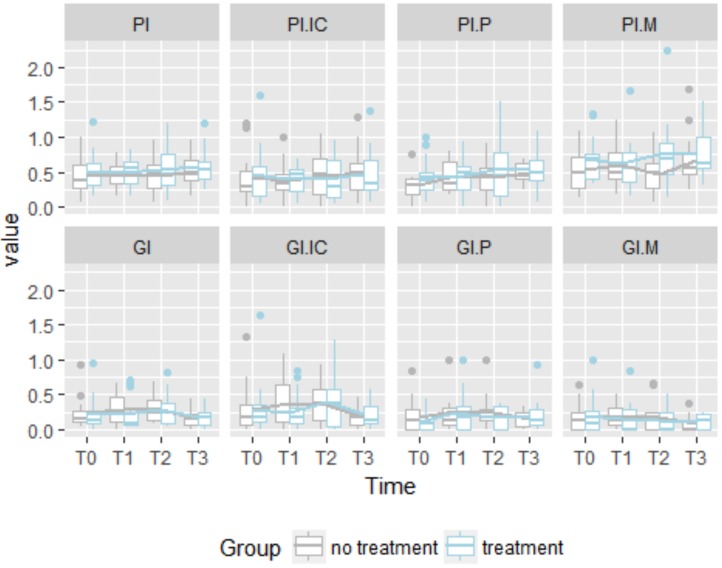
Boxplot of Plaque Index (Incisiors-canines, premolars, molars) and Gingival Index Index (Incisiors-canines, premolars, molars) per group and time period.

 Table 1 shows the means and standard deviations for each measure, treatment and time period. Here is evident that in some case variance is not equal across time periods. This is pointed out in the Fig. 1 that shows the distributions of all the measures for each treatment across the time.

See that the range of the boxplots are not the same denoting a sort of heterogeneity of variance. The lines highlight the means of the two different treatments, the cleaning system with the waterjet is marked in lightblue while the stardard cleaning system in grey. In general, the global means of Plaque Index and Gengival Index, respectively PI and GI, do not seem to have different trends across the time but in T3 when we found a decreasing trend for the GI metric. For what concerns the PI, in the sample with the waterjet treatment, we can see that PI mean at T0 is higher in the group with treatment than the mean in the sample without this treatment. Then, in the other periods we notice the trends are constant for both the groups. If we consider the GI, we can see a higher deviation between the two groups in T1 but it reduces in T2 and T3. In GI.IC we found the same trend behavior described before, but taking into account GI.P, GI.M we do not find any significant deviation between the groups across the time periods according to a descriptive point of view. The same occurs with PI.IC, PI.P but PI.M, in fact we can see that the mean value of PI.M of the group with waterjet treatment is higher than the other group especially in T2 and T3.

In Fig. 1 we can see the value of all the metrics for each patient, while the average trends are highlighted with a thicker line. Note that the individual trends assume different patterns.

To test these descriptive conclusions from an inferential point of view, we performed the pairwise t-test for each metric in order to find out whether the mean is significantly different across the time periods in each group.  Table 2 shows the p-values that are all higher the 0.05 but four comparisons. In fact, we can find a p-value lower than 0.05 for what concerns the comparison between the mean of the group with treatment in T3 and T1 of the measure PI.M. Another significant difference between means has been found in the group without treatment comparing the metric PI.P in T3 and T0. Other two significant differences between means have been found in the group without treatment comparing the metric GI of T3 versus T1 and T3 versus T2. Finally, the last significant difference between means has been found in the group without treatment comparing the metric GI.C in T3 and T2. Thus, statistically significant differences across the same group have been found in the comparison between T3 and other time period only.

**Table 2:**
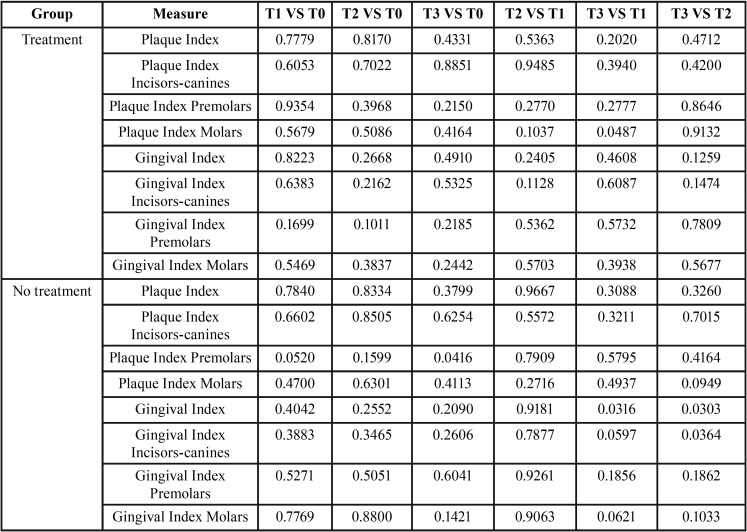
*p*-values of pairwise comparison among time periods of Plaque and Gingival indexes per group.

 Table 3 shows the results of the statistical tests to figure out whether the means of the two groups are significantly different in each time periods. Also, in this case there is no significant difference given that all the *p*-values are higher than 0.05.

**Table 3:**
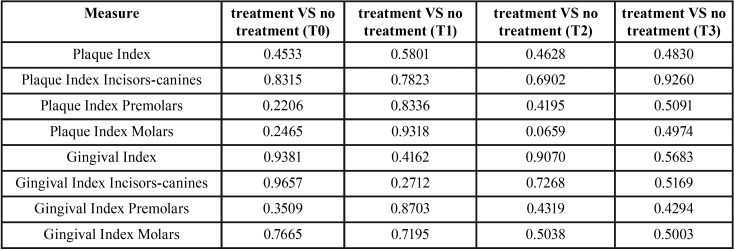
*p*-values for comparing the group means of Plaque and Gingival indexes in each time period.

## Discussion

Different authors studied the effect of dental water jet and found not only a significant reduction of gingival inflammation and bleeding scores but also a reduction of bacterial population in different patient’s sample: patients affected by diabetes (5), patients under periodontal treatment (6-9), patients with crowns and/or bridges on natural elements or implants (10,11) and after maxillo-facial operations (12). Literature regarding the use of dental water jet on orthodontic patients is actually limited since only one randomised clinical trial has been published. The aim of Sharma *et al.* study was to assure the efficacy of dental water jet and a particular orthodontic tip on plaque and bleeding scores on an adolescence population wearing a fixed appliance (13). The authors of this study found a statistically significant improvement at a two- and four-weeks follow-up. There is not any study that analyse these parameters on the long term of an orthodontic treatment. For this reason we decided to record the scores at 1 month and later at 3 and 6 months to obtain a more complete view on the temporal progression of the home oral hygiene in an orthodontic patient sample. Results obtained in our study showed no significance difference between the treated and the control sides evidencing a non-significance efficacy of dental water jet in reducing plaque and bleeding scores in the long term during an orthodontic treatment. In the study published in 2008, however, all three groups showed a significance reduction at 14 and 28 days. In particular those patients which used both the manual toothbrush and dental water jet were recorded a plaque score 2.34 times better than the control group at 2 weeks and 3 times at 4 weeks. Authors reported also a slight improved bleeding score that resulted 1.07 more efficient than the floss and 1.39 more efficient than the toothbrush alone. These results may be determined by the short follow-up and the by following the patient with a particular and strict hygienic control. Their results are incredibly encouraging since patients show a clear improvement also in comparison with the baseline before the fixed appliance bonding. In fact, different studies in literature evidence that the application of a multibracket appliance determines a worsening of both plaque and bleeding indexes (14-17). In a recent study published by Boke *et al.* in 2014, authors evaluated the periodontal status before, during and after the orthodontic treatment and found a significant plaque accumulation and gingivitis in the period between the baseline and debonding (18). Similar results were found in a study published by Zachrisson *et al.* (19). These authors reported that, even if patient have maintained a good oral hygiene, they developed mild to moderate gingivitis in two months from the appliance bonding. Similar results were published by Liu *et al.*, evidencing that a fixed orthodontic appliance determine biofilm and plaque accumulation with a significant increase of plaque and gingival index in the very first period after bonding (20).

In the present study, authors found a linear trend for both plaque and gingival index on the side treated with dental water jet and the control one at T0, T1, T2 and T3. In this sample it does not emerge the traditional worsening of orthodontic patient as soon as the appliance bonding. These encouraging results cannot be due only to the dental water jet use since no statistical significant difference between the two sides emerged. Authors found a possible explanation of these results in the “novelty effects” according to which introducing a new technology or a new appliance, people tend to perform better for an increased attention given to the activity they are performing (21). In this case, introducing a new appliance such as the dental water jet may have increased the attention given to the proper home oral hygiene by the patients. The role of the dental hygienist is fundamental for a healthy periodontal care before, during and after the orthodontic treatment with particular attention in teaching how to perform an efficient daily care and in motivating them (22,23).

## Conclusions

The results obtained from this randomised controlled trial evidenced that the dental water jet does not improve significantly the efficacy of home oral hygiene in orthodontic patients wearing a multi-bracket fixed appliance.

The patient sample did not show the typical worsening of plaque and bleeding indexes (24,25), maintaining the baseline score for the whole follow-up. The positive improvement of plaque and gingival indexes found in this study is probably due to the strict dental hygienist follow-up in combination to the novelty effect of using the dental water jet domiciliary, increasing the global attention on oral hygiene.

## Clinical relevance

The scientific rationale of the study was to provide evidence on the possible usefulness of the dental water jet during the orthodontic treatment for the plaque and inflammation control.

The principal finding was that the dental water jet does not improve significantly the efficacy of home oral hygiene in orthodontic patient if compared with traditional brushing. Patients on the other hand did not show the typical worsening of the oral hygiene condition after the application of multibracket appliance.

A practical implication is that clinicians can use the novelty effect to increase patients’ attention to oral hygiene. Furthermore a strict control from the dental hygienist can determine encouraging results.
